# Cutaneous Leishmaniasis of the Eyelid: A Case Report

**DOI:** 10.1155/2013/214297

**Published:** 2013-10-10

**Authors:** Jamshid Ayatollahi, Ali Ayatollahi, Seyed Hossein Shahcheraghi

**Affiliations:** ^1^Infectious and Tropical Diseases Research Center, Shahid Sadoughi University of Medical Sciences, Avenue Ebnesina, Safaeeyeh, Yazd 8915887857, Iran; ^2^Department of Optometry, School of Rehabilitation, Shahid Beheshti University of Medical Sciences, Tehran 1985717443, Iran

## Abstract

Cutaneous leishmaniasis is endemic in certain areas of Iran, and in recent years, there has been an increase in the number of reports for rare and new forms of cutaneous leishmaniasis. We describe one unusual clinical form of cutaneous leishmaniasis. In a 27-year-old man, who noted a pimple on the upper eyelid 4 months before. The lesion was nodular and measured 1 cm × 1 cm in diameter. A diagnosis of eyelid cutaneous leishmaniasis was made, and treatment was started with intramuscular meglumine antimonate. He showed a dramatic response, and the lesion almost completely disappeared.

## 1. Introduction

Cutaneous leishmaniasis is a common protozoal disease and an important public health problem in Iran [1]. Cutaneous Leishmaniasis is a zoonotic disease caused by the *Leishmania* spp., and transmission occurs through the bite of a female sandfly infected with *Leishmania* parasites [2]. The *Leishmania* spp. may produce several clinical syndromes. Cutaneous leishmaniasis is the most common form, and the initial lesion is a nodule at the bite site by an infected sandfly. Fever or pain is not a feature of ulcer. We describe an unusual site of infection, eyelid cutaneous leishmaniasis. The patient was from the Yazd province, which is in the center of Iran.

## 2. Case Report

**Figure 1:**
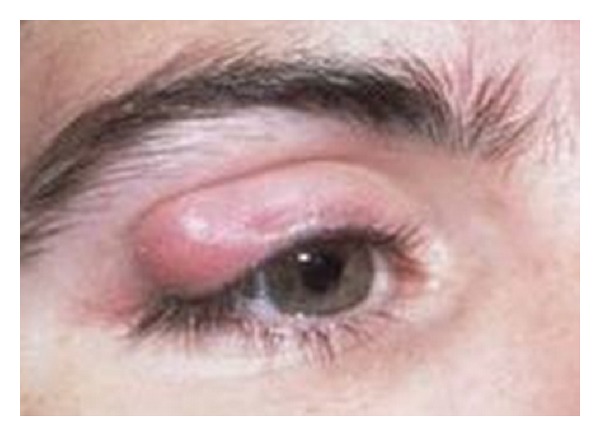
The lesion of eyelid on right eye.

 A 27-year-old man from center of Iran noted a small pimple on eyelid 4 months before presentation. The eyelid lesion enlarged slowly. It was as a well-defined, firm, nontender, and subcutaneous skin-colored nodule. The lesion measured 1 cm × 1 cm in diameter (Figure 1).

The lesion had been enlarging slowly and had slightly erythematous, raised borders and was crusty and painless. CBC (complete blood cell count), ESR (erythrocyte sedimentation rate), CRP (C-reactive protein), and blood chemistries gave results within normal limits. Sample culture of the lesion grew *Staphylococcus aureus*. Various treatments with cephalexin, cloxacillin, amoxicillin-claulanate, and steroids failed to heal the lesion. These treatments were performed for 10 days Although there was cutaneous leishmaniasis in the patient's residence, and also because lack of response to treatment, cutaneous leishmaniasis or basal cell carcinoma was proposed as a differential diagnosis for him.

**Figure 2:**
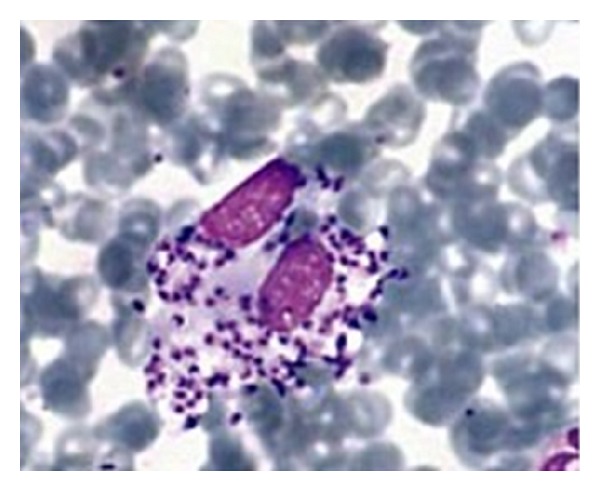
Smear shows leishman bodies.

A slit-skin smear was taken from the edge of the nodule before a biopsy could be taken. Giemsa stain of the sample showed numerous leishman bodies within the macrophages (Figure 2), and the diagnosis of cutaneous leishmaniasis was confirmed. The patient was treated with meglumine antimonate (glucantime) in a dose of 20 mg/kg/body weight intramuscularly for 20 days, with slow but definite improvement of the lesion. The patient reported mild arthralgias and mild myalgias but otherwise tolerated the meglumine antimonate well. After one year of followup, there has been no relapse or new lesion.

## 3. Discussion

Cutaneous leishmaniasis is caused by different species of the protozoan *Leishmania* with different clinical features [3]. Many of the clinical presentations are typical and present no diagnostic difficulties [4]. The most commonly involved sites are exposed areas. The face is the usual site in the cutaneous leishmaniasis; however, the low incidence on the eyelid is attributed to the frequent movements of the eyelid, which deter the insect from biting the skin in this region [5].

Eyelid cutaneous leishmaniasis has rarely been reported [5–7]. Our patient had been mistakenly treated for bacterial infection on several occasions, and ultimately the diagnosis was based on the presence of amastigotes in lesion.

Differential diagnosis for cutaneous leishmaniasis includes secondarily infected insect site, eczema, malignancy, cutaneous tuberculosis, and other cutaneous disorders, early detection of the infection is necessary in order to start effective treatment and prevention from inappropriate prescribing of drugs.

In our knowledge, eyelid cutaneous leishmaniasis is unusual, having only been described in few cases [5–9]. In an endemic area and in a traveler from endemic area, it is necessary for the physician to be aware that any atypical lesion should be surveyed for cutaneous leishmaniasis and treated with meglumine antimonate if is necessary [10].
